# Treatment outcomes for isoniazid-resistant tuberculosis under program conditions in British Columbia, Canada

**DOI:** 10.1186/s12879-017-2706-0

**Published:** 2017-09-04

**Authors:** Kamila Romanowski, Leslie Y. Chiang, David Z. Roth, Mel Krajden, Patrick Tang, Victoria J. Cook, James C. Johnston

**Affiliations:** 1 Provincial Tuberculosis Services, BC Centre for Disease Control, Vancouver, BC Canada; 2 BC Centre for Disease Control Public Health Laboratory Medicine, University of BC, Vancouver, BC Canada; 3 Department of Pathology, Sidra Medical and Research Center, Doha, Qatar; 4 Division of Respiratory Medicine, University of BC, Vancouver, BC Canada

**Keywords:** Mycobacterium tuberculosis, INH resistance, Tuberculosis treatment

## Abstract

**Background:**

Every year, over 1 million people develop isoniazid (INH) resistant tuberculosis (TB). Yet, the optimal treatment regimen remains unclear. Given increasing prevalence, the clinical efficacy of regimens used by physicians is of interest. This study aims to examine treatment outcomes of INH resistant TB patients, treated under programmatic conditions in British Columbia, Canada.

**Methods:**

Medical charts were retrospectively reviewed for cases of culture-confirmed INH mono-resistant TB reported to the BC Centre for Disease Control (BCCDC) from 2002 to 2014. Treatment regimens, patient and strain characteristics, and clinical outcomes were analysed.

**Results:**

One hundred sixty five cases of INH mono-resistant TB were included in analysis and over 30 different treatment regimens were prescribed. Median treatment duration was 10.5 months (IQR 9–12 months) and treatment was extended beyond 12 months for 26 patients (15.8%). Fifty six patients (22.6%) experienced an adverse event that resulted in a drug regimen modification. Overall, 140 patients (84.8%) had a successful treatment outcome while 12 (7.2%) had an unsuccessful treatment outcome of failure (*n* = 2; 1.2%), relapse (*n* = 4; 2.4%) or all cause mortality (*n* = 6; 3.6%).

**Conclusion:**

Our treatment outcomes, while consistent with findings reported from other studies in high resource settings, raise concerns about current recommendations for INH resistant TB treatment. Only a small proportion of patients completed the recommended treatment regimens. High quality studies to confirm the effectiveness of standardized regimens are urgently needed, with special consideration given to trials utilizing fluoroquinolones.

## Background

Tuberculosis (TB) remains a leading cause of infectious disease death worldwide, with 10.4 million new cases and 1.8 million deaths annually [1]. While the incidence of TB is decreasing globally, recent surveys indicate that drug-resistant TB exists in virtually every location examined [1]. Isoniazid (INH) is an important first-line agent for the treatment of TB given its potent early bactericidal activity and extensive evidence base as a first line therapy for drug susceptible TB [2, 3] Unfortunately, resistance to INH has been detected in 1 in 3 incident TB cases in Eastern Europe and 1 in 7 incidence TB cases in all other regions [4]. Indeed, over 1 million people develop INH resistant TB globally each year.

Systematic reviews and meta-analyses have confirmed that INH resistance reduces the probability of treatment success and increases the risk of acquiring resistance to other first line drugs including rifampin, thereby increasing the risk of multidrug resistant TB (MDR-TB) [5, 6]. Despite the global burden of INH resistance and reduced probability of treatment success, the optimal regimen and duration of treatment for INHR-TB remains controversial. In the setting of known first line drug susceptibility results, the World Health Organization (WHO) treatment guidelines [7] recommend using rifampin (RIF), pyrazinamide (PZA) and ethambutol (EMB) for 6–9 months. The Canadian Tuberculosis Standards 7th edition and American Thoracic Society offer similar recommendations: 6–9 months of RIF, PZA, and ETH with the potential addition of a fluoroquinolone (FQN) [8, 9]. However, limited published evidence support these regimens and all three bodies acknowledge further research is required in this area.

In the absence of robust evidence for specific treatment regimens, a wide variety of treatment regimens are used by treating physicians [10–12]. Given the increasing prevalence of INH resistance, the clinical efficacy of regimens used by physicians is of interest. In this study, we aimed to identify and described the variations in treatment regimens for patients with confirmed INH mono-resistance and report on outcomes when treated under routine programmatic conditions in British Columbia (BC), Canada.

## Methods

### Study setting and data source

BC is a Canadian province of 4.6 million people with a TB incidence of 6.3 per 100, 000 population [13]. The BC Centre for Disease Control (BCCDC) is a centralized public health agency that maintains a TB registry of all active TB cases across BC through notification by public health partners and routine reporting from the centralized provincial mycobacteriology laboratory and provincial pharmacy [13].

### Data collection

From the BCCDC TB registry, we identified all cases of culture-confirmed, INH mono-resistant TB from November 1, 2002 to December 31, 2014. Cases were excluded if treatment duration was ≤30 days*,* or if end of treatment outcomes were unavailable at the time of data extraction**.** Patient demographics, comorbidities, medical history, bacteriologic information, radiologic data, detailed treatment information, adverse events, treatment outcomes, and post-treatment follow-up information were extracted through individual chart review from the BCCDC TB registry.

### Specimen processing and drug-susceptibility testing

The BACTEC 460-radiometric method (Becton Dickinson, Franklin Lakes, NJ) or subsequently the BACTEC MGIT 960 System (Becton Dickinson) were used to determine drug susceptibilities of *Mycobacterium tuberculosis* isolates at the BCCDC Public Health Laboratory. Drugs and their critical concentrations for resistance were as follows: INH at 0.1 μg/mL and 0.4 μg/mL*,* rifampin at 1.0 μg/mL*,* ethambutol at 5.0 μg/mL, and streptomycin at 1.0 μg/mL in accordance with Clinical and Laboratory Standards Institute recommendations [14]. INH resistance was classified as either low level or high level, when there was a > 1% growth of *M. tuberculosis* in the presence of 0.1 μg/mL or 0.4 μg/mL of INH, respectively. INH mono-resistance was defined as resistance to INH alone or INH plus streptomycin, without evidence of resistance to other first line anti-TB drugs. Patients with resistance to INH and one other first line anti-TB drug were excluded from analysis.

### Outcome measures and definitions

Treatment outcomes were defined as per the Canadian TB Standards 7th edition [9].

- *Cure:* culture-negativity at the completion of treatment.
- *Treatment complete:* a complete course of active TB therapy without culture confirmation of cure or evidence of failure at the end of the treatment course.
- *Treatment non-completion (CTBS term: default):* treatment stopped for ≥2 months before completing ≥80% of doses.
- *Treatment failure:* positive sputum culture after ≥4 months of treatment or two positive sputum cultures in different months during the last 3 months of treatment, even if final culture was negative and no further treatment is planned.
- *Death*: mortality from any cause.
- *Recurrence*: disease recurrence after initial cure or treatment complete, without genotypic evidence of the same organism by 24-loci Mycobacterial Interspersed Repetitive Unit-Variable Number of Tandem Repeats (MIRU-VNTR) testing [15].
- *Relapse*: disease recurrence after initial treatment cure or complete, with genotypic evidence of the same organism by MIRU-VNTR testing. For analytic purposes *recurrence* was considered the same as *relapse*.
- *Acquired drug resistance:* new or additional resistance to one or more of the TB drugs received in the setting of failure or relapse.

For the purpose of this study, a *successful outcome* included patients meeting the definition of *Cure* or *Treatment Completed* while an *unsuccessful outcome* included patients meeting the definition of *Treatment Failure*, *Acquired Drug Resistance, Death* or *Relapse*. Relapse was assessed using the BCCDC TB registry.

### Data analysis

Statistical analysis was performed using SPSS (V.23) and R (V.3.2.2), with a level of significance in reference to a 2-tailed, type 1 error (*P* value) set as <0.05. Univariate analysis was performed using the X^2^ test or Fisher exact test for dichotomous variables and Mann-Whitney for continuous variables.

## Results

### Baseline characteristics

In total, 184 cases of culture confirmed INH mono-resistant TB were identified from the BCCDC TB registry; 165 of the 184 cases (89.7%) were included in our analysis. Reasons for study exclusion included duration of treatment ≤30 days (*n* = 10) and patient transfer (*n* = 9). Of the 10 patients who were treated for ≤30 days, 5 patients died, 3 patients transferred, and 2 patients with extra-pulmonary disease had their medications discontinued due to medical complications.

The baseline characteristics of the included patients are reported in Table 1. Of the 165 included patients, 103 (62.4%) were males and median age was 46 years (IQR 32.5–61 years). The majority of patients (*n* = 155; 69.7%) were born in a country with a TB incidence >30 per 100,000 population; 127 (77.0%) patients had no prior history of TB treatment. The most common comorbidity was diabetes mellitus (*n* = 19; 11.5%), followed by the use of an immune suppressive medication (*n* = 13; 7.9%). Six patients (3.6%) were identified as HIV positive. Of the 106 cases (64.2%) with pulmonary involvement, 73 (59.3%) were sputum acid-fast bacillus smear positive, and 50 (40.7%) had at least one cavitary lesion present on chest x-ray. Concentrations for INH resistance were reported for all 165 patients. Eighty one patients (49.1%) had low-level INH resistance alone while 84 (50.9%) had both low and high-level INH resistance.

**Table 1:** Demographic and clinical characteristics

Characteristic	Total patients *n* = 165
Sex (*n*, %)	
Male	103 (62.4)
Female	62 (37.6)
Age, years (IQR)	46 (32.5–61)
Region of origin (*n*, %)	
Canadian born	44 (26.7)
Foreign born	
TB incidence >30 per 100,000	115 (69.7)
TB incidence of ≤30 per 100,000	5 (3.0)
Unknown country of birth	1 (0.6)
Year of diagnosis	
2002–2006	37 (22.4)
2007–2010	56 (33.9)
2011–2014	72 (43.6)
Co-morbidities (*n*, %)	
Diabetes mellitus	19 (11.5)
Immune suppressive medication	13 (7.9)
Malignancy	7 (4.2)
HIV positive	6 (3.6)
Chronic kidney disease	2 (1.2)
Prior history of TB treatment	
No prior TB treatment	127 (77.0)
Prior TB treatment	24 (14.5)
Prior TB treatment unknown	14 (8.5)
Disease site	
Pulmonary	106 (64.2)
Extra-pulmonary	42 (25.5)
Pulmonary and extra-pulmonary	17 (10.3)
Extent of pulmonary disease^a^	
Baseline positive AFB smear	73 (59.3)
Cavitary lesions in chest radiography	50 (40.7)
Isoniazid resistance (*n*, %)	
INH, both high and low level resistance	84 (50.9)
INH, only low level resistance	81 (49.1)

^a^The denominator used to calculate the percentage for *Extent of pulmonary disease* was based on cases with pulmonary involvement (*n* = 123)

### Treatment regimens

Within our cohort, over 30 different regimens were prescribed; we combined them based on treatment duration, length of PZA therapy, and the presence of fluoroquinolone therapy. Treatment regimens are described in Table 2. A total of 89 patients (53.9%) were prescribed a regimen of RIF, EMB and PZA. Of those, 41 patients (24.8%) only received PZA during the intensive phase while 48 patients (29.0%) received PZA throughout the entire treatment course.

**Table 2:** Treatment regimen composition and characteristics

Treatment Regimen *Intensive phase/Continuation phase*	Total patients (*n * = 165)^a^
6 to <9 Months (*n*, %)	
(H)RZE/(H)RE	8 (4.8)
(H)RZE/(H)RZE	6 (3.6)
(H) RZEQ/REQ	6 (3.6)
Other	1 (0.2)
≥ 9 to ≤12 Months (*n*, %)	
(H)RZE/(H)RE	32 (19.4)
(H)RZE/(H)RZE	35 (21.2)
(H)RZE(Q)/(H)REQ	14 (8.5)
(H)RZE(Q)/(H)RZEQ	10 (6.1)
(H)RE/(H)RE	4 (2.4)
Other	17 (10.3)
> 12 Months (*n*, %)	
(H)RZE/(H)RE	1 (0.2)
(H)RZE/(H)RZE	6 (3.6)
(H)RZE(Q)/(H)REQ	4 (2.4)
Other	15 (9.1)

*I* isoniazid, *P* pyrazinimide, *R* rifampin, *E* ethambutol, *Q* fluoroquinolone, *S* streptomycin

^a^6 (3.6%) patients were on treatment for <6 months due to treatment non-completion or death

Median length of treatment was 10.5 months (IQR 9–12 months). Treatment was extended beyond 12 months for 26 patients (15.8%). 12 (7.3%) patients received extended treatment due to an adverse events (AEs) resulting in treatment modification, 6 (3.6%) patients received extended treatment due to treatment noncompliance, while another 6 (3.6%) had extended treatment due to physician preference, and 2 (1.2%) because of extensive disease. Patients with extended treatment where more likely to have their regimen supplemented with a FQN when compared to patients who completed ≤12 months of treatment (65.4% vs. 31.7%, *p* = 0.002).

In total, 56 patients (33.6%) experienced an AE that resulted in a drug regimen modification. Of those, 14 (8.9%) patients experienced a second AE for a total of 70 events. Of the 70 AEs, 65 could be attributed to a single drug, and 5 could not. Frequency of AEs can be seen in Table 3. PZA was discontinued for 21(12.7%) patients due to 11 cases of drug-induced hepatitis, 7 due to rash and 3 due to nausea and vomiting. Ten patients (6.0%) were intolerant to RIF. Occurrence of AEs was not significantly associated with any demographic or clinical characteristics in univariate analysis (data not shown).

**Table 3:** Frequency of adverse drug reactions

	Total patients *n* = 165
Number of patient who required drug regimen modification due to adverse reaction (*n*, %)	56 (33.3)
Number of patients who stopped treatment due to adverse events (*n*, %)	2 (1.21)
Total number of adverse events	70
**Adverse event**	**Number of events**
Drug induced hepatitis	20
Rash	12
Nausea and vomiting	11
Tendonopathy	8
Blurred vision	6
Optic Neuropathy	2
Arthralgia	2
Other	9

### Treatment outcomes

Treatment outcomes are summarized in Table 4. At the end of treatment, 144 patients (87.3%) experienced treatment completion or cure and therefore, met the definition of treatment success. Two patients (1.1%) experienced treatment failure and no patients acquired drug resistance in the setting of treatment failure. All cause mortality during treatment was reported for 6 (3.6%) patients; 2 patients died from lung cancer, 2 from respiratory failure, 1 from kidney failure, and 1 from cardiac arrest. No deaths resulted from side effects of anti-TB drugs.13 patients (7.8%) were classified as treatment non-completion, including 2 patients who stopped treatment early due to adverse drug reactions; treatment non-completion was not significantly associated demographic or clinical characteristics in univariate analysis.

**Table 4:** Clinical treatment outcomes

Treatment outcomes	Total patients (*n* = 165)
End of treatment outcomes (*n*, %)	
Treatment success at end of treatment	144 (87.3)
Treatment non-completion	13 (7.8)
Death	6 (3.6)
Failure	2 (1.2)
Treatment outcomes in follow-up (*n*,%)	
Relapse	4 (2.4)
Acquired drug resistance	0 (0)
Primary study outcomes	
Successful outcome^a^	140 (84.8%)
Microbiologically confirmed unsuccessful outcome^b^	12 (7.2%)

^a^A successful outcome included patients meeting the definition of *Cure* or *Treatment Completed* without *Relapse*

^b^An unsuccessful outcome included patients meeting the definition of *Treatment Failure*, *Acquire Drug Resistance, Death,* or *Relapse*

The median follow-up duration post treatment was 8 months (IQR 2–18.5 months). Of the 144 patients with treatment success at the end of treatment, 4 patients (2.4%) experienced relapse. MIRU-VNTR confirmed identical patterns in 2 of 4 relapse strains, while 2 strains were not typed by MIRU-VNTR. In the 4 patients with relapse, 2 patients were treated with 9HRZE, 1 patient was treated with 12HRZE, and 1 was treated with 2HRE/4HR. No demographic or clinical characteristics were associated with patient relapse in univariate analysis (data not shown). No patients acquired drug resistance in the setting of relapse.

Overall, 140 patients (84.8%) met the study definition of a successful outcome while 12 patients (7.2%) met the definition of an unsuccessful outcome. Treatment success rates were similar in patients with high vs. low-level INH resistance profiles (90.7% vs. 93.4%; *p* = 0.547). No statistically significant difference was observed between patients with successful outcomes versus microbiologically confirmed unsuccessful outcomes despite multiple comparisons between treatment regimens and patient characteristics (Table 5). Multivariate analysis was not performed due to the small number of patients with the combined endpoint of a microbiologically confirmed unsuccessful outcome.

**Table 5:** Comparison of characteristics with favourable versus unfavourable outcomes

Characteristic (*n*, %)	Successful outcome^a^ (*n* = 140)	Unsuccessful outcome^b^ (*n* = 12)	*P* value
Patient characteristics			
Co-morbidity (HIV, DM, Malignancy, CKD, immune suppressive medication)	36 (25.7)	3 (25.0)	1.00
Smear positive disease^c^	64 (60.4)	5 (50.0)	0.523
Cavitary disease^c^	41 (38.7)	5 (50.0)	0.515
Prior TB treatment^d^	19 (14.4)	2 (22.2)	0.623
Resistance profile			
High level INH resistance	69 (49.3)	7 (58.3)	0.547
Treatment regimen			
> 2 months pyrazinamide	66 (47.1)	7 (58.3)	0.456
> 9 months rifampin	69 (49.3)	3 (25.0)	0.106
≥ 4 effective drugs in intensive phase	25 (17.9)	3 (25.0)	0.464
Fluoroquinolone containing regimen	52 (37.1)	4 (33.3)	0.793

^a^A successful outcome included patients meeting the definition of *Cure* or *Treatment Completed* without *Relapse*

^b^An unsuccessful outcome included patients meeting the definition of *Treatment Failure*, *Acquire Drug Resistance, Death,* or *Relapse*

^c^The denominator used to calculate percentage for smear positive disease was based on those with pulmonary involvement and primary study outcome (successful outcome *n* = 106; unsuccessful outcome *n* = 10)

^d^Missing data for 8 successful outcomes and 1 unsuccessful outcome

## Discussion

In this study, we found that 84.4% of patients experienced a successful treatment outcome under programmatic conditions in BC. Our treatment outcomes are consistent with those from other INH resistant treatment cohorts in high resource, low incidence settings [10–12, 16]. In resource-limited settings, where detailed individual level drug susceptibility results are not always accessible, the proportions of patients experiencing unsuccessful outcomes are often much higher. For example, in an INH resistance treatment cohort in rural South Africa, 15% of patients experienced treatment failure, of which 61% progressed to MDR-TB [17].While the differences in treatment outcomes may be reflective of differences in patient and health care resources rather than regimen efficacy, it is worth noting that in studies where resistance was detected early and drug regimens were modified, a higher proportion of patient’s experienced successful outcomes [11, 12]. This suggests that outcomes of treatment in INH resistant disease may be related to early detection of resistance and individualized therapy.

Within our cohort, over 30 different treatment regimens were prescribed; regimens were adjusted based on DST patterns, adverse events, severity of disease, and physician preference. Regimens were also often extended. However, similar to other studies [12], 12.7% of patients in our study were unable to tolerate PZA and the high incidence of drug toxicity suggests that new treatment regimens are needed to improve INH-resistant TB treatment outcomes.

In our cohort, 3 cases of relapse that occurred in patients who completed on a 9–12 month regiment of only RIF, PZA and EMB. Meanwhile, no cases of relapse developed in the 61 (37.0%) patients receiving an FQN-containing regimen. This finding, while not statistically significant, is in line with growing evidence suggesting that supplementation with FQNs may strength treatment regimens among patients with INH-resistant disease [16, 18, 19]. In a recent retrospective analysis of treatment outcomes with FQN containing regimens [18], the authors concluded that INH resistant pulmonary TB is associated with improved outcomes when FQNs are added to standard treatment regimens (97.3% vs. 84.6%, *P* = 0.007).

In 2011, *Jenkins* et al. reviewed the global burden and trends of INH resistant TB using surveillance data reported to the WHO [4]. The authors concluded that in several geographically disparate settings, the number of new TB cases with INH resistance is increasing [4]. This is consistent with routine surveillance data from BC and Canada, which show an increase in the prevalence of INH resistance [13]. As the burden of INH resistant disease increases, the need for a standard, cost-effective, evidence-based treatment regimen becomes more pressing. Prolonged, individualized courses for INH resistant TB are not practical, and would be difficult to implement in resource limited settings where the highest INH resistance burden exists [1, 4]. The WHO currently recommends two different treatment regimens for INH resistance; one for countries with an assumed ‘high’ level of INH resistance and another based on a setting of known first line drug susceptibility results [7, 20]. Unfortunately, the controversies regarding both of these treatment regimens outweigh the evidence [5, 6] and highlight the need for controlled trials to validate specific standardized recommendations.

Our study had several limitations. The most important was our inability to control for potential confounding variables and effect modification. Instead, we had to rely exclusively on univariate analysis for our analysis of outcomes due to the small sample size of unsuccessful outcomes. Whereas prior studies have identified characteristics of different treatment regimens associated with improved treatment outcomes, such as extending PZA duration [21], addition of fluoroquinolones [18], or use of four effective drugs in the intensive phase [5], these findings were not statistically evident in our cohort, possibly the result of the relatively small combined endpoint.

We were also limited by the variation in the composition and duration of treatment regimens. There was great variation in the composition and duration of treatment regimens making it impossible to identify superior or inferior regimens given the type of study and the sample size. Additionally, within each regimen group, there may have been clinically important differences that we could not account for. Highly heterogeneous treatment regimens are a widely recognized to be present in clinical practice, particularly when evidence behind treatment regimens is scarce [10–12, 16]. The wide variety of treatment regimens presented here reflects the uncertainty of clinicians in appropriate treatment of INH resistant disease.

Despite these limitations, our study raises important concerns about the currently recommended treatment regimens for INH resistant TB. It highlights the need for high quality studies to firmly establish standardized treatment regimens, with special consideration given to trials that utilize fluoroquinolones. Currently, there is little evidence and much controversy regarding the recommended treatment regimens, and given the global burden of INH resistance, solid evidence validating the various recommendations for treatment is urgently needed.

## Abbreviations

AE: Adverse event
DST: Drug susceptibility testing
EMB: Ethambutol
FQN: Fluoroquinolone
INH: Isoniazid
MDR-TB: Multi-drug resistant TB
PZA: Pyrazinamide
RIF: Rifampin
TB: Tuberculosis
